# Modelling testing and response strategies for COVID-19 outbreaks in remote Australian Aboriginal communities

**DOI:** 10.1186/s12879-021-06607-5

**Published:** 2021-09-08

**Authors:** Ben B. Hui, Damien Brown, Rebecca H. Chisholm, Nicholas Geard, Jodie McVernon, David G. Regan

**Affiliations:** 1 Kirby Institute, UNSW Sydney, Wallace Wurth Building, Sydney, NSW Australia; 2 The Peter Doherty Institute for Infection and Immunity, The Royal Melbourne Hospital and The University of Melbourne, Melbourne, VIC Australia; 3 Centre for Epidemiology and Biostatistics, Melbourne School of Population and Global Health, The University of Melbourne, Melbourne, VIC Australia; 4 Department of Mathematics and Statistics, School of Engineering and Mathematical Sciences, La Trobe University, Melbourne, VIC Australia; 5 School of Computing and Information Systems, The University of Melbourne, Melbourne, VIC Australia; 6 Victorian Infectious Diseases Reference Laboratory Epidemiology Unit, The Peter Doherty Institute for Infection and Immunity, The Royal Melbourne Hospital and The University of Melbourne, Melbourne, VIC Australia

**Keywords:** COVID-19, Indigenous health, Outbreaks, Quarantine, Patient Isolation, Households, Family and Household

## Abstract

**Background:**

Remote Australian Aboriginal and Torres Strait Islander communities have potential to be severely impacted by COVID-19, with multiple factors predisposing to increased transmission and disease severity. Our modelling aims to inform optimal public health responses.

**Methods:**

An individual-based simulation model represented SARS-CoV2 transmission in communities ranging from 100 to 3500 people, comprised of large, interconnected households. A range of strategies for case finding, quarantining of contacts, testing, and lockdown were examined, following the silent introduction of a case.

**Results:**

Multiple secondary infections are likely present by the time the first case is identified. Quarantine of close contacts, defined by extended household membership, can reduce peak infection prevalence from 60 to 70% to around 10%, but subsequent waves may occur when community mixing resumes. Exit testing significantly reduces ongoing transmission. Concurrent lockdown of non-quarantined households for 14 days is highly effective for epidemic control and reduces overall testing requirements; peak prevalence of the initial outbreak can be constrained to less than 5%, and the final community attack rate to less than 10% in modelled scenarios. Lockdown also mitigates the effect of a delay in the initial response. Compliance with lockdown must be at least 80–90%, however, or epidemic control will be lost.

**Conclusions:**

A SARS-CoV-2 outbreak will spread rapidly in remote communities. Prompt case detection with quarantining of extended-household contacts and a 14 day lockdown for all other residents, combined with exit testing for all, is the most effective strategy for rapid containment. Compliance is crucial, underscoring the need for community supported, culturally sensitive responses.

**Supplementary Information:**

The online version contains supplementary material available at 10.1186/s12879-021-06607-5.

## Background

The SARS-CoV-2 pandemic continues to cause significant morbidity and mortality worldwide, disproportionately affecting vulnerable and disadvantaged groups such as those of lower socio-economic status, or with comorbidities [1]. Protecting such groups must be a priority. As of mid-2021, Australia remains in a favourable position compared with much of the world and although several outbreaks have led to regional lockdowns, COVID-19 case numbers since January 2020 have totalled only around 30 450, with 910 deaths [2]. No cases of community transmission have yet occurred in remote Australian Aboriginal and Torres Strait Islander communities.

Aboriginal and Torres Strait Islander peoples (hereafter respectfully referred to as ‘Aboriginal’), however, are significantly more vulnerable to severe COVID-19 than the non-Aboriginal population due to a high prevalence of comorbidities that are associated with more severe clinical outcomes [3]. The incidence of chronic respiratory diseases is 1.2 times higher than for non-Aboriginal Australians, type 2 diabetes 3.3 times higher, and chronic kidney disease 3.7 times higher [4]. SARS-CoV-2 transmission is likely to be even more intense within remote communities due to crowded housing, larger family sizes, inadequate hygiene facilities, and residence across multiple dwellings (4–7). These communities are also further from specialist health services, with SARS-CoV-2 tests needing to be transported, thereby resulting in delays to diagnosis and treatment. Previous influenza outbreaks in these communities have underscored their vulnerability. During the 2009 H1N1 pandemic, hospital and ICU admissions for Aboriginal people were 12 and 5 times higher, respectively, than for non-Aboriginal Australians [5]. Similarly, First Nations Americans have suffered from COVID-19 rates 3.5 times that of white Americans, and mortality rates that are almost double [6]. The consequences of overcrowding and disadvantage have also been demonstrated in Singapore, where migrant workers in overcrowded dormitories suffered from infection rates of up to 20% [7].

In Australia, protection of remote Aboriginal communities was prioritised early, with strict movement controls, within designated biosecurity zones, established in consultation with communities [8]. The Aboriginal and Torres Strait Islander Advisory Group on COVID-19 (IAG), co-chaired by the Department of Health and the National Aboriginal Community Controlled Health Organisation, provides evidence-based and culturally safe guidance for COVID-19 preparedness and response to the government and other key stakeholders, with a view to locally led adoption of recommendations within each community [9]. This group liaises with peak national health advisory bodies on COVID-19 and commissioned the work that we present here to help inform optimal public health response strategies in remote settings.

This study presents a novel exploration of COVID-19 control interventions in remote Aboriginal communities in Australia, which are vulnerable to COVID-19 due to the underlying comorbidities, and with infection expected to transmit quickly due to overcrowding and dynamic household structure that extends beyond single dwellings. The model output was also used to shape the COVID-19 outbreak response policy for these communities. A report outlining the key results of this work and recommendations is publicly available from the Australian Government Department of Health [10].

## Methods

We compare plausibly implementable non-pharmaceutical-based strategies in a remote Aboriginal community, examining the impact of alternative scenarios in an outbreak response, including: initial delays with testing; differing definitions of case-contacts and consequent quarantine strategies; community-wide lockdowns; and exit testing strategies. We assume a wholly susceptible, unvaccinated population as the COVID-19 vaccine uptake rate in remote communities has so far been low [11].

A participatory approach was employed throughout this study. All SARS-CoV-2 outbreak response scenarios explored were designed through iterative engagement between the academic investigators, the IAG, and other public health end-users to ensure cultural sensitivity, and to maximise the relevance and uptake of findings.

An individual-based model, repurposed from a framework developed to examine dynamics of sexually transmitted infections in remote Australia, is used to explicitly represent each community member [12]. Community sizes comprising 100, 500, 1000 or 3500 people are modelled, with results presented here focusing on communities of 1000 people but noting key differences. The full detail of the model is provided in the Additional file 1: Appendix S1, with the key features and assumptions for this analysis highlighted in the following sections.

### Population assumptions

The population of each community comprises individuals with SARS-CoV2 infection status tracked and updated daily. Transmission of infection can occur if there is contact between an infectious individual and a susceptible individual. Contacts can occur between individuals who share the same dwelling on a particular day (household contacts), and less frequently, between individuals who do not share the same dwelling on a particular day (community contacts).

The population household structure used is adopted from a study investigating the effects of a household-focused prophylaxis intervention on an outbreak of an influenza-like illness in Australian Aboriginal communities conducted by Chisholm et al.[13]. This study has shown the importance of accounting for community structure and related mixing patterns for infection and control dynamics. Interventions were found to be less effective in communities where individuals are assigned to multiple rather than single dwellings, particularly in populations with a high level of household crowding, and when the risk of transmission within households greatly exceeded that in the wider community. In the model used here, individuals have family connections across multiple dwellings and each individual’s total time “at home” is distributed between a main dwelling (core; 66% of the time), second dwelling (regular 23% of the time), and third dwelling (on/off; 9% of the time). The remaining 2% of time at home is spent at a randomly allocated dwelling. These percentages are based on a framework for Australian Indigenous mobility proposed to reflect observations of occupancy from a single dwelling over time in a remote Aboriginal community in central Australia as described by Musharbash [14]. The frequency of contact, and therefore likelihood of transmission, is higher between individuals within the same dwellings. Section 3 of the Additional file 1: Appendix S1 provides a summary of household distribution and contact rates.

### Epidemic assumptions

**Fig. 1:**
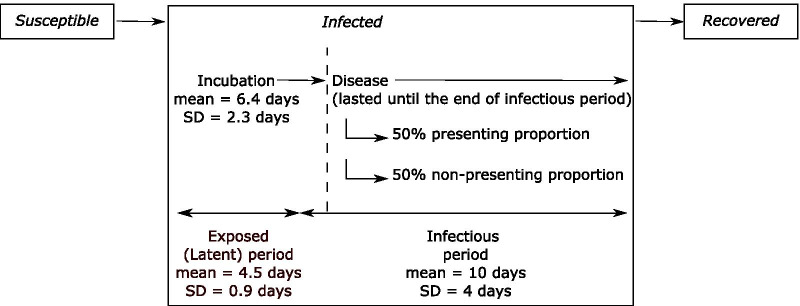
Susceptible, Infected, Recovered Model Assumptions. After exposure to SARS-CoV-2 a proportion of susceptible individuals become infected, entering the incubation phase before proceeding to the disease phase. 50% of individuals in the disease phase are assumed to spontaneously present to clinical services, the ‘presenting proportion’. The remaining ‘non-presenting proportion’ (those with minor, no or unrecognised symptoms or who avoid health services due to fear or stigma) will only be identified through active case finding and testing efforts as part of the public health response. We assume infectiousness commences 48 h prior to onset of symptoms (if they occur) and persists until resolution of symptoms. While we do not explicitly split out asymptomatics from the non-presenting proportion, we conservatively assume that they are as infectious as individuals with symptoms

The disease model follows a susceptible, exposed, infectious, recovered paradigm and captures the time to onset of infectiousness (Latent period) and symptoms (Incubation period) as illustrated schematical in Fig. 1. We assume infectiousness commences 48 h prior to symptom onset on average [15] and ceases with symptom resolution. Table A1 of the Additional file 1: Appendix S1 summarises the key transmission parameters. The basic reproduction number *R*_*0*_ was calibrated, through adjustment to transmission probability per contact, to centre around 5, based on similar contexts [16–18] and allowing for enhanced mixing anticipated in overcrowded households [19–21] (Sect. 2 of the Additional file 1: Appendix S1; the results for additional analyses conducted under the assumption of smaller values for *R*_*0*_ of 2 and 3 are contained in Sect. 7.4 of the Additional file 1: Appendix S1). We conservatively assume that only 50% of infected patients will self-present for testing, due either to minimal/no/unrecognised symptoms, fear/anxiety, or stigma.

### Intervention assumptions

The impact of a multi-layered public health response is assessed following identification of the index case. *Cases* (those who test positive for SARS-CoV-2) are assumed to be isolated immediately and effectively. *Contacts* of cases, as variously defined below, are quarantined alone and assumed to be completely separated from others.

#### Contact definitions

**Fig. 2:**
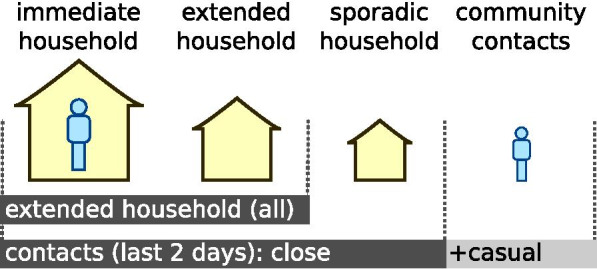
Definitions of contacts. Household-based contacts include the index case’s immediate and extended households defined by dwelling. History-based contact tracing relies on active contact tracing for the preceding 2 days, including household (close) contacts and community (casual) contacts. The immediate household consists of the index case’s place of residence at the time of diagnosis. The extended household comprises the index case’s immediate household in addition to their other dwellings (i.e., core, regular and on/off)

Two broad strategies for contact definition are assessed as per Fig. 2. For household-based, we define *immediate* household contacts as those who share the same dwelling at the time of tracing; *extended* household contacts are those who share other dwellings that a case frequently inhabits (i.e., main, second and third dwelling as described previously). For history-based contact tracing, contacts are those identified over the prior 2 days (close and casual).

#### Case isolation and quarantine

Apart from the ‘No response’ scenario, we assume each identified case of positive SARS-CoV-2 infection will be immediately placed in isolation for 10 days in accordance with National guidelines [22]. A clearance test, if applied, is scheduled to occur on the 8^th^ day of isolation. Contacts of the case are also placed in quarantine for 14 days, with a clearance test, if applied, scheduled to occur on the 12th day of quarantine.

We assume both isolation and quarantine are ideal, and that an individual cannot transmit or be infected with SARS-CoV-2 while in isolation or quarantine. Table A4 in the Additional file 1: Appendix S1 provides a summary of contact possibilities for individuals in isolation and quarantine.

#### Testing

Initial testing of individuals for SARS-CoV-2 occurs following clinical presentation, or after identification as a contact. We assume a 2-day delay between a test being conducted and initiation of the public health response. We also assume 100% test sensitivity. The impact of subsequent testing is examined under the following scenarios:

- *Entry* testing of all contacts when commencing quarantine;
- *Clearance* testing prior to release from *quarantine* for all contacts (on day 12 of 14, assuming a 2-day delay between the test being conducted and diagnosis becoming available);
- *Clearance* testing prior to release from *isolation* for all cases (on day 8 of 10, assuming a 2-day delay between the test being conducted and diagnosis becoming available);
- *Clearance* testing prior to release from *lockdown*;

Positive diagnoses at any point are treated as new cases. For non-clearance tests, positive diagnoses trigger a further round of contact tracing with subsequent isolation and quarantine.

#### Lockdown of community

Concurrent with the quarantining of contacts, the impact of a 14-day lockdown of all households within a community is assessed. We assume lockdown is triggered at the first diagnosis of COVID-19 in the population. In this study, the first diagnosis is made when the first infected individual in the population seeks a test (i.e., due to symptoms). Under lockdown, individuals remain in their core dwelling and can mix with other residents of that dwelling, but *not* with residents of other households or the wider community. Table A4 in the Additional file 1: Appendix S1 details the contact possibilities for an individual in lockdown compared with those for an individual in isolation or quarantine.

### Scenarios investigated

We present the results of this study in three main sections. First, we investigate the impact of delays in initial case finding over a range of population sizes by assessing the number of individuals infected with SARS-CoV-2 at the time when one, two and five positive diagnoses have been made. Focusing on a population size of 1000, we then assess the impact on COVID-19 outbreaks (cumulative infections, person-days in quarantine, number of tests conducted) under case isolation and testing and/or quarantine of infected contacts. Finally, we assess the impact on COVID-19 outbreaks when community lockdown is introduced in addition to the testing and quarantining of infected household contacts. For the latter we also consider the mitigating impact of lockdown on delays in the initiation of interventions, and the impact of compliance and community size on the effectiveness of lockdown.

Table A6 in the Additional file 1: Appendix S1 listed all scenarios investigated in this study along with their key epidemic characteristics, including the duration of the outbreak, the peak prevalence and the size of the outbreak.

## Results

### Impact of delays to initial case identification

For our analyses, we assume a scenario in which an initial case enters the community while pre-symptomatic and is detected only on subsequent self-presentation and testing. This introduces a delay during which this index case can transmit infection to others. The number of infected individuals likely present in the community by the time the first case, the first two cases and the first five cases are identified is summarised in Table 1. Figure A2 of the Additional file 1: Appendix S1 summarises projected numbers if a lower (than 50%) proportion of cases self-present for testing.

**Table 1:** Impact of delay to initial case identification

Population size	One case identified		Two cases identified		Five cases identified	
	Current infected individuals	Cumulative infected individuals	Current infected individuals	Cumulative infected individuals	Current infected individuals	Cumulative infected individuals
100	9 (5, 16)	32 (15, 47)	18 (11, 25)	52 (38, 65)	37 (28, 46)	84 (71, 90)
500	7 (2, 15)	29 (9, 55)	20 (12, 30)	73 (50, 104)	48 (37, 63)	162 (134, 207)
1000	6 (3, 14)	27 (10, 59)	19 (11, 27)	72 (46, 100)	50 (33, 68)	184 (131, 235)
3500	7 (4, 11)	22 (9, 42)	18 (11, 25)	66 (44, 105)	49 (35, 67)	187 (144, 247)

Scenarios are shown for a range of community sizes, summarising the number of currently infected individuals and the cumulative number of infected individuals present by the time that the initial one, two or five cases are identified. Medians with interquartile ranges (in brackets) are reported from 100 simulations

### Impact of definition of contacts, and quarantine strategies

**Fig. 3:**
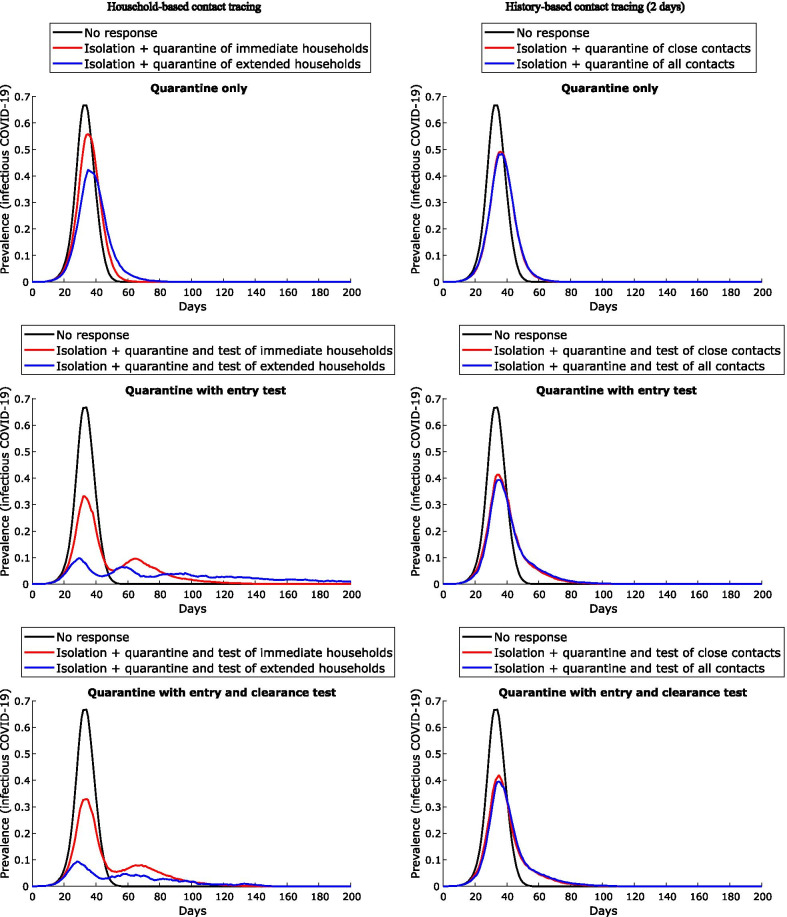
Impact of different contact tracing strategies: epidemic curves for a community of 1000 people, comparing the household-based tracing strategy, at left, with the history-based tracing strategy (for the prior 2 days) at right. Lines represent the median value and shaded areas the interquartile ranges from 100 simulations

In the absence of entry and clearance testing, the extended household-based contact tracing and quarantine strategy results in a peak infection prevalence of approximately 40%, versus 50% for the history-based quarantine strategy (Fig. 3, upper panels). The addition of *entry* testing to quarantine reduces the peak infection prevalence for the extended household-based strategy to approximately 10%, versus 40% for the history-based strategy (middle panels). Adding both *entry and clearance* testing results in a small additional benefit to the extended household strategy (largely in the reduction of outbreak duration), but no substantial benefit to the history-based strategy.

The impact of clearance testing with various quarantine strategies on total infection numbers (i.e., not just peak prevalence) is greatest for the extended household-based contact tracing approach (Table 2). In all other strategies, more than 90% of the community are ultimately infected, with or without testing. For extended household quarantine *without* clearance testing, 83% are infected, ~87,000 person-days spent in quarantine and > 4000 tests performed. The addition of clearance testing results in ~66% being infected, fewer person-days in quarantine (~51,000) but more tests (13,551), making it the most effective strategy.

**Table 2:** Impact of clearance testing on contact tracing and quarantine strategies for a community of 1000 people

Strategy	No clearance testing			Clearance testing undertaken		
	Total cumulative infections	Quarantine person-days (first year)	Total tests performed during outbreak (first year)	Total cumulative infections	Quarantine person-days (first year)	Total tests performed during outbreak (first year)
No response	999 (999–999)	N/A	447.0 (435.5, 458.0)	999	N/A	N/A
Quarantine of *immediate* household contacts (with case isolation)	922.0 (907.5, 936.5)	29,595.5 (28,101.5–31,175.0)	1957.5 (1867–2027)	922.5 (905.0, 933.0)	22,500.5 (21,469.0–23,306.0)	7526.0 (7336–7743)
Quarantine of *extended* i.e. all household contacts (with case isolation)	831.5 (751.0, 871.0)	86,825.0 (70,334.5–97,662.5)	4042.5 (3463–4305)	655.0 (267.5, 821.0)	50,958.0 (13,511.5, 67,786.0)	13,551.5 (4929.5, 16,729.5)
Quarantine of *close* contacts based on history (past 2 days)	937.0 (929.0, 945.0)	10,776.5 (9551.5–11,564.5)	1530.5 (1441–1586)	930.5 (917.0, 939.5)	9445.5 (8541.5, 10,191.5)	4673.5 (4549.5, 4780.5)
Quarantine of *all* contacts based on history (past 2 days)	930.0 (917.0, 941.0)	11,887.0 (11,180.0–12,831.5)	1614.5 (1550–1667)	919.0 (904.5, 931.5)	10,662.0 (9718.0, 11,768.5)	4842.5 (4741.0, 4957.0)

Size of outbreak (total cumulative infections), quarantine person-days (per 1000 population), and total tests performed during an outbreak are shown. Medians are reported, with interquartile ranges (in brackets) from 100 simulations. Note that ‘infections’ refers to all individuals with SARS-CoV-2 infection (whether tested and known to health services or not), whereas ‘cases’ refers to those with SARS-CoV-2 infection who have tested positive, i.e., have been identified. For all scenarios shown in the table, entry testing (which leads to further rounds of contact tracing, isolation and quarantine) is conducted upon entry to quarantine

### Impact of community lockdown

**Fig. 4:**
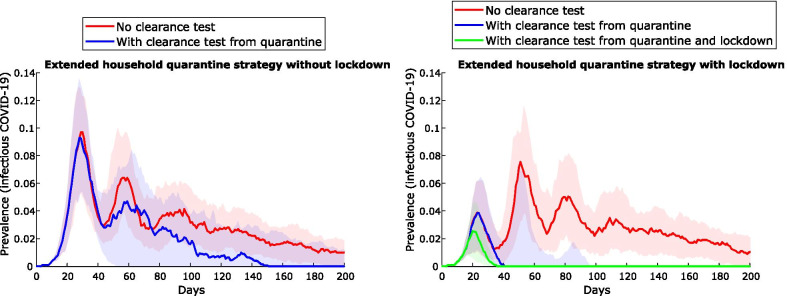
Impact of community lockdown on the extended household-based quarantine strategy, for a community of 1000 people. The panel at left shows epidemic curves for the non-lockdown scenario (a composite of outputs from Fig. 3), with and without clearance tests from quarantine. The panel at right shows these scenarios with lockdown. Entry testing is assumed to occur for all individuals. Lines represent the median value and shaded areas the interquartile ranges from 100 simulations

Building on the extended-household quarantine strategy, the impact of *lockdown on all remaining households* (i.e., non-quarantined households) is shown to reduce both epidemic peak and duration – particularly if clearance testing is undertaken (Fig. 4). Clearance testing from quarantine and lockdown is the most effective strategy to avert subsequent waves of infection in the community (green line). Entry testing is assumed to occur for all these scenarios.

Lockdown with clearance testing is also the most effective strategy to reduce *total* cumulative infections when applied alongside the extended household quarantine strategy with clearance testing (Table 3). Without any clearance testing (top row), lockdown alone has little impact on total infections (> 800), quarantine person-days (> 85,000), or tests (~4000). Adding clearance testing to quarantine only (middle row) results in fewer infections with lockdown added (89 versus 655), similar quarantine person-days (~5000), and far fewer tests (1402 versus 13,551). Undertaking clearance testing for both lockdown and quarantine (bottom row) results in only 35 infections in total, fewer quarantine person-days, and ~2500 tests—the optimal strategy.

**Table 3:** Impact of lockdown and extended household quarantine, combined with various testing strategies, for a community of 1000 people

Strategy	No lockdown			Full lockdown		
	Total cumulative infections	Quarantine person-days (first year)	Total tests performed during outbreak (first year)	Total cumulative infections	Quarantine person-days (first year)	Total tests performed during outbreak (first year)
Quarantine of *extended* household contacts (no clearance testing)	831.5 (751.0, 871.0)	86,825.0 (70,334.5—97,662.5)	4042.5 (3463—4305)	829.0 (712.0, 866.5)	85,283.0 (69,397.0, 92,022.5)	3927.5 (3434.5, 4156.0)
Quarantine of *extended* household contacts with clearance testing from quarantine	655.0 (267.5, 821.0)	50,958.0 (13,511.5, 67,786.0)	13,551.5 (4929.5, 16,729.5)	88.5 (20.0, 432.5)	5253.5 (1660.5, 24,531.0)	1402.0 (344.5, 7564.0)
Quarantine of *extended* household AND clearance testing for entire community	N/A	N/A	N/A	35.0 (9.0, 62.5)	3469.0 (1431.5, 5602.5)	2498.0 (2169.5, 2823.5)

The effect on size of outbreak (total cumulative infections), quarantine person-days (per 1000 population), and total tests performed during outbreak are shown. Medians are reported, with interquartile ranges (in brackets) from 100 simulations. Note that quarantine of the entire community without lockdown is not investigated here as it would involve the testing and/or quarantine of individuals who have no exposure history and this is not recommended under current public health guidelines

### Impact of delays in initiation of interventions on effectiveness of lockdown

**Fig. 5:**
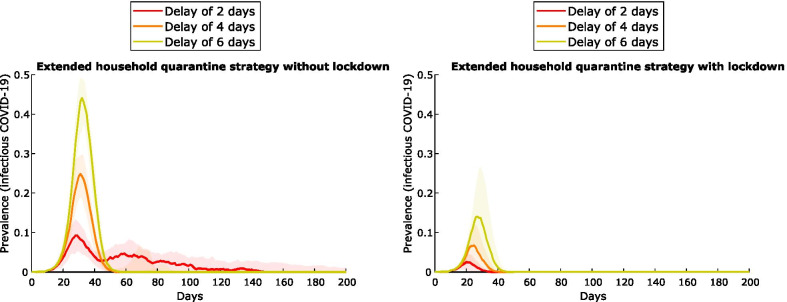
Impact of lockdown on outbreak control, comparing delays in the response following testing of index case. Epidemic curves shown for the extended household quarantine scenario in a community of 1000 people, with entry and clearance testing. Initial outbreak response following the identification of the index case is delayed by 2, 4 or 6 days; the no-lockdown scenario is shown at left, with lockdown at right. Median values and interquartile ranges (shaded) from 100 simulations are shown

The effect of delays between the identification of cases and implementation of interventions is mitigated by the addition of lockdown (Fig. 5). For the extended household quarantine scenario, increasing the delay from 2 to 4 days in the absence of a lockdown, causes infection prevalence to increase from < 10% to ~25%, and to ~45% with a 6-day delay (left panel). The addition of lockdown results in a peak prevalence of < 15%, even with a 6-day delay to implementation (right panel). In this latter case, the median outbreak size is restricted to approximately 200 individuals, which is smaller than the median outbreak size (486 + individuals) under scenarios with shorter delays but without lockdown (Table A-6 of the Additional file 1: Appendix S1).

### Impact of compliance with lockdown

**Fig. 6:**
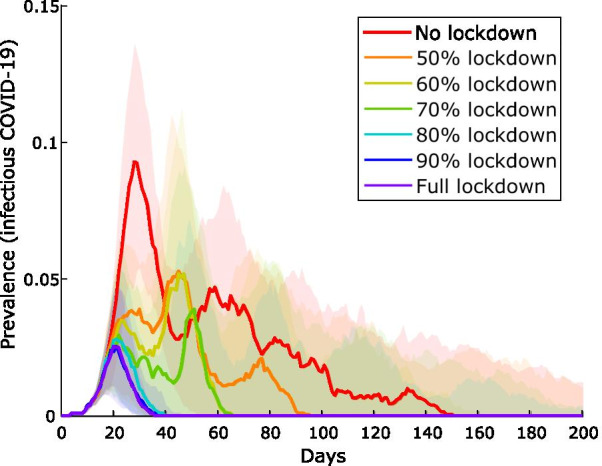
Impact of compliance with lockdown on a community of 1000 people. Epidemic curves for the extended household quarantine strategy (with entry and clearance testing), with various levels of individual compliance with community lockdown. Median values and interquartile ranges (shaded areas) from 100 simulations shown

Loss of epidemic control occurs even in the optimal strategy (lockdown alongside the extended household quarantine strategy, with entry and clearance testing) when compliance for individuals with lockdown falls below 80% (Fig. 6).

### Impact of community size on the effect of lockdown

**Fig. 7:**
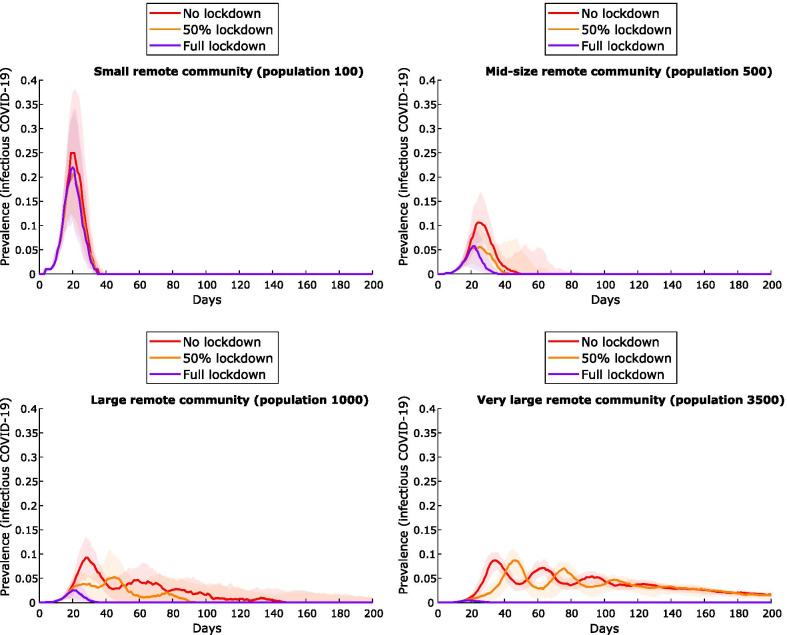
Impact of lockdown on communities of varying size. Epidemic curves for the extended household quarantine scenario (with entry and clearance testing), with perfect lockdown; lockdown with 50% compliance; and no lockdown. Median values (lines) and interquartile ranges (shaded areas) from 100 simulations shown

For small communities of 100, lockdown has little additional impact as under the extended household quarantine strategy, most of the population is already quarantined due to interconnectedness of a large proportion of the community at the time lockdown is triggered (Fig. 7). For communities of 500, lockdown reduces peak prevalence from ~10% under the extended household quarantine strategy to ~5%. Greatest benefit is seen in very large communities (3500), where peak prevalence is reduced from ~10% to less than 1%, and subsequent waves of infection are suppressed.

## Discussion

Prompt case finding is essential to prevent a SARS-CoV-2 outbreak in a remote Aboriginal community. A high transmission propensity, due to interconnected and often crowded households, means that in an unmitigated scenario the majority of the community would be rapidly infected. By the time early cases are identified, active infections in the community may be up to ten-fold higher. This is especially apparent for smaller communities due to the high degree of interconnectedness of individuals, as compared to larger communities where the number of households is larger and distinct but separate clusters of households are more likely to form. We assume only half of all infected patients will self-present to health services for testing, due to absent, unrecognised or minimal symptoms, fear, or stigma. This may be an overestimate, but there is evidence that pre-symptomatic transmission may contribute > 40% of SARS-CoV-2 transmission exists [15, 23]. This non-presenting proportion may not be detected using a passive case finding approach, although a high prevalence of other co-morbidities may result in non-COVID related presentations resulting in opportunistic case detection. Higher non-presenting proportions would lead to poorer mitigation in all scenarios, while interventions will have greater impact if this proportion is higher than assumed here (see Additional file 1: Appendix S1).

Of the contact tracing strategies assessed, quarantining *extended household* members (residents of all dwellings used by the case) is the most effective strategy for constraining the initial outbreak, reducing peak prevalence from 60 to 70% to ~ 10% (Fig. 3) for a community of 1000 individuals. However, large numbers of people must be quarantined for extended periods and outbreaks are predicted to resurge when community mixing resumes, with overall community attack rates exceeding 80% (Table 3). Clearance testing modestly reduces this attack rate to 65%. Lockdown of all non-quarantined households for 14 days, concurrent with this quarantine strategy, results in the greatest likelihood of definitive outbreak control. Peak prevalence of the initial outbreak is less than 5%, and the overall attack rate less than 10%. Clearance testing from lockdown further improves control, preventing subsequent waves of infection following the release of individuals with undetected infection (Fig. 4): overall infections are constrained to < 5% with clearance testing, versus > 80% without. In contrast, without clearance testing and/or additional changes to response strategies, subsequent waves of infection are highly likely following the release of large numbers of susceptible individuals from quarantine and/or lockdown restrictions resulting in recrudescence of infection among susceptible individuals. Clearance testing from the lockdown strategy also results in fewer tests due to prompt suppression, fewer person-days in quarantine, and is effective in mitigating outbreaks with delays of up to 6 days (Fig. 5). Larger communities benefit most from lockdown, with the effect dampened in smaller communities (100–500) by the large proportion already in quarantine. Compliance with lockdown must be at least 80–90%, or epidemic control will be lost. While this is an ambitious target given the lockdown compliance rate has been observed to be closer to 50–60% in Australian cities [24], our consultation with stakeholders suggested that a higher compliance rate is possible for remote communities due to smaller population size and physical isolation. Furthermore, with COVID-19 vaccination now available in remote communities of Australia, it is likely that a lower compliance rate in lockdown will be required to control outbreaks.

Our findings are consistent with recent guidelines for a ‘contain and test’ strategy developed by Central Australian health organisations [8], which acknowledge that symptom-based case identification will be insufficient, and endorse active case finding and lockdown with multiple rounds of voluntary testing. Analyses of SARS-CoV-2 outbreaks overseas also support the effectiveness of lockdowns. In the Italian town of Vo, researchers concluded that a 14-day lockdown reduced transmissibility of infections (including asymptomatic) by 82–98% [25]. Lockdowns in Wuhan contributed to a significant decrease in spread [26], and an analysis of French data suggested that over 80% of potential COVID-19 deaths were averted by their lockdowns [27]. Recent modelling from the UK, examining the impact of delays with testing and contact tracing, suggests that if cumulative delays exceed 3 days for these processes, control of an outbreak is unlikely [28].

The participatory process employed between this study’s investigators, the IAG, and other public health end-users throughout, has allowed for direct feedback of our findings and incorporation into IAG guidelines [9], and collaborative development of plain-language messaging for health providers and community members. Our findings support the suggestion that prompt case finding and a rapid public health response upon first diagnosis will be critical for effective control [29], which in our context, can be facilitated by having access to decentralised point-of-care testing (e.g., *GeneXpert*). Local planning and preparation should occur in advance, and must involve community members to ensure cultural appropriateness, local support and community control. Early patient presentation should be encouraged, and testing, contact tracing and isolation/quarantine guidelines and facilities clarified. The extensive public health response required to achieve best outcomes necessitates prior preparedness planning to ensure that the significant logistical and human resources support needed can be rapidly mobilised. Throughout an outbreak, community trust must be preserved in order to maximise compliance; in particular, the historical context and consequent sensitivities regarding enforced lockdowns in remote Aboriginal communities must be kept foremost in mind in the design and implementation of such strategies.

## Limitations

In developing our model, simplifying assumptions due to limited observational data regarding population structure and mixing were necessary. Other than household structure, ‘real-world’ mixing opportunities such as schools and workplaces have not been explicitly included. Assumptions regarding transmission dynamics are derived from non-Aboriginal populations, but where possible we have erred on the side of caution. For example, the high *R*_*0*_ to which the model is calibrated is based on early estimates from Wuhan and amplified to reflect the propensity for intense transmission in remote households. However, as shown in the additional analyses described in Sect. 7.4 of the Additional file 1: Appendix S1, majority of our findings still apply for smaller *R*_*0*_ of 2 and 3. We also note that at the time of writing, the Delta variant of SARS-CoV-2, which is believed to have an effective reproduction number close to double that of the previous variants [30], has reached Australia and has sparked outbreaks in several jurisdictions resulting in strict lockdowns in Sydney, Melbourne and Adelaide [31]. It is therefore entirely possible that our assumed *R*_*0*_ of 5 for remote communities is close to or even underestimates the real *R*_*0*_ should the Delta variant reach these communities. We also assume perfect sensitivity and specificity of testing throughout the infectious period. Morbidity and mortality outcomes have not been estimated in this model, or the anticipated demand on health resources (testing requirements aside).

We assume that cases in isolation and contacts in quarantine will have no contact with others (i.e., will not transmit SARS-CoV-2). This may not be possible to achieve for every remote communities, but this assumption was based on stakeholder input from the Northern Territory, Australia, where suitable facilities have been made available for use to many communities by the resources sector. By representing this ideal we assess the maximum effectiveness of these measures and demonstrate the added value of lockdown. We also assume a wholly susceptible, unvaccinated population, as less than 5% of the total Australian population was fully vaccinated as of mid-2021[32], with this proportion likely to be lower in remote communities [11]. However, the expansion of Australia’s vaccine rollout, with prioritisation of remote areas and at-risk populations, will increase the proportion of the population that is vaccinated over time.

## Conclusions

Remote Australian Aboriginal and Torres Strait Islander communities have the potential to be severely impacted by COVID-19, due to factors favouring increased transmission and disease severity. Our modelling affirms the need for early case detection as multiple secondary infections are likely already present by the time an index case is identified. Quarantining of extended household contacts, together with 14-day community-wide lockdown with clearance testing, are the most effective strategies in limiting the outbreak.

## Supplementary Information

**Additional file 1: Appendix S1.** COVID-19 remote model.

## Data Availability

Project name: Package_RMP. Project home page: The code for the model is available on https://github.com/The-Kirby-Institute, with raw data available upon request. Operating system(s): Platform independent. Programming language: Java.

## Abbreviations

COVID-19: Coronavirus Disease 2019
H1N1: Influenza A virus subtype H1N1
IAG: Aboriginal and Torres Strait Islander Advisory Group on COVID-19
ICU: Intensive care units
SARS-CoV-2: Severe acute respiratory syndrome coronavirus 2
